# Primary Retroperitoneal Paraganglioma Simulating a Pancreatic Mass: A Case Report and Review of the Literature

**DOI:** 10.1155/2010/645728

**Published:** 2010-12-06

**Authors:** Guillermo Sangster, Daniel Do, Carlos Previgliano, Benjamin Li, Delecia LaFrance, Maureen Heldmann

**Affiliations:** ^1^Department of Radiology, Louisiana State University Health Sciences Center Shreveport, 1501 Kings Hwy. Shreveport, LA 71130-3932, USA; ^2^Department of Surgery, Louisiana State University Health Sciences Center Shreveport, 1501 Kings Hwy. Shreveport, LA 71130-3932, USA; ^3^Department of Pathology, Louisiana State University Health Sciences Center Shreveport, 1501 Kings Hwy. Shreveport, LA 71130-3932, USA

## Abstract

Paragangliomas are extra-adrenal tumors of the autonomic nervous system and may be found within the skull base, neck, chest, and abdomen. When presenting within the abdominal cavity, they may arise as a primary retroperitoneal neoplasm and can mimic vascular malformations or other conditions related to specific retroperitoneal organs such as the pancreas, kidneys, or adrenals. Retroperitoneal paragangliomas are mostly benign with good prognosis; however, they can present with abdominal pain, palpable mass, or hypertensive episodes. Patients should be initially evaluated with catecholamine levels, followed by computed tomography or magnetic resonance imaging to locate the primary lesion. Surgical excision remains the mainstay of treatment, although advanced disease and proximity to vital organs can make excision difficult or impossible. This case report describes a patient who initially underwent work up for a suspected pancreatic head mass which was discovered to be a retroperitoneal paraganglioma by frozen section.

## 1. Introduction

Paragangliomas are extra-adrenal tumors of the autonomic nervous system and may be found within the skull base, neck, chest, and abdomen. When presenting within the abdomen, they may arise as a primary retroperitoneal neoplasm and can be mistaken for other retroperitoneal tumors, including those arising from the pancreas. This case report describes a patient who initially underwent work up for a suspected pancreatic head mass which was discovered to be a paraganglioma by frozen section. In addition, a review of current literature was performed summarizing radiographic and clinical findings associated with retroperitoneal paragangliomas.

## 2. Case Report

**Figure 1:**
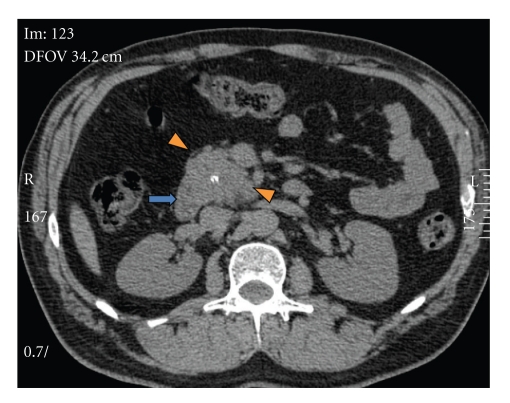
Axial noncontrast CT abdomen demonstrates a retroperitoneal soft tissue lesion (arrowheads) with lobulated margins and a central calcification. Prominent vessels are noted around the lesion. The second duodenal portion contacts the mass (arrow).

**Figure 2:**
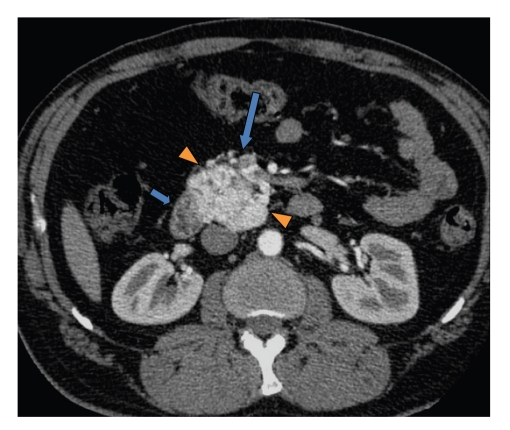
Axial contrast enhanced CT abdomen shows a hypervascular retroperitoneal mass (arrowheads) with enlarged surrounding vessels (large arrow). No cleavage plane is noted between the duodenum and the mass (short arrow). Also the mass contacts the inferior vena cava (IVC).

**Figure 3:**
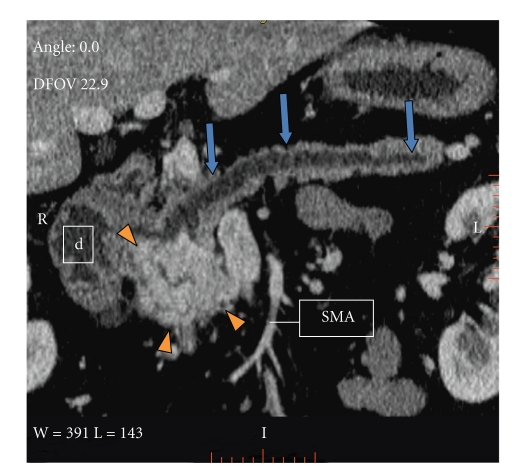
Contrast enhanced CT abdomen. Curved multiplanar reconstruction demonstrates an intimal relation of the retroperitoneal paraganglioma, and the proximal pancreas, duodenum (d), and IVC. Pancreatic body and tail show parenchymal atrophy and pancreatic ductal dilatation (arrows). SMA: superior mesenteric artery.

This is a 50-year-old white male who originally presented in 2004 with complaints of epigastric abdominal pain. The patient's past medical history was significant for hypertension, 30-pack per-year history of tobacco use, occasional ETOH use, diabetes, hyperlipidemia, and regular marijuana use. Workup at that time revealed elevated amylase and lipase levels, a dedicated gallbladder ultrasound was unremarkable, and the final diagnosis of thiazide-induced pancreatitis was made. The medication was discontinued, laboratory values normalized, and the patient was discharged. The patient presented again in 2005 with similar complaints and lab abnormalities. Upon questioning, the patient stated he continued his thiazide medication since prior discharge; the thiazide was again discontinued. Following clinical improvement the patient was discharged and switched to an ACE inhibitor for blood pressure management. In the outpatient clinical setting, the patient continued to have unresolved epigastric abdominal discomfort despite unremarkable amylase and lipase values. In light of this, a computed tomography (CT) scan of the abdomen and pelvis was performed in early 2006 demonstrating a hypervascular retroperitoneal mass intimately related to the pancreas (Figures 1, 2, and 3). A positron emission tomography (PET) scan performed 7/2006 demonstrated intense FDG uptake in the region of the pancreatic head without locoregional nodal or distant metastatic disease. Fine needle aspiration of the mass was suggestive of poorly differentiated carcinoma. These findings prompted surgical consultation and a Whipple procedure was planned for resection of the possible pancreatic head mass. In 8/2006 surgical exploration revealed a retroperitoneal mass situated inferior to the uncinate process which extended to the infrarenal abdominal aorta. The mass demonstrated close involvement with the inferior vena cava and superior mesenteric vessels. Direct involvement of the pancreas and adrenals was not appreciated. Given the unusual location of the mass as well as its aortic proximity, resection was deferred. 

**Figure 4:**
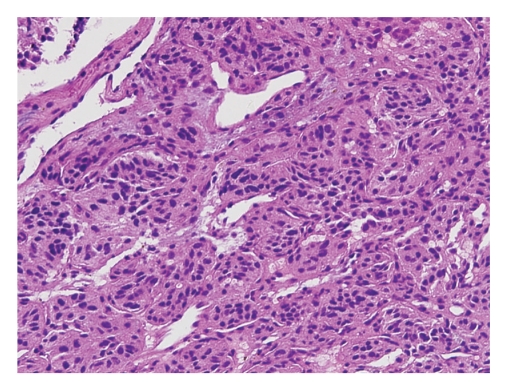
Paraganglioma composed of dual cell population arranged in characteristic nested pattern “zellballen” (20x).

**Figure 5:**
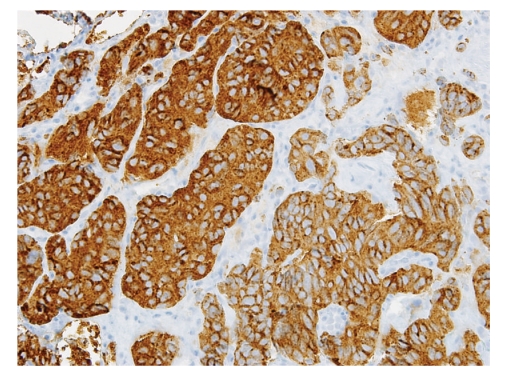
Synaptophysin (brown immunohistochemical stain) confirms neuroendocrine origin, supporting the diagnosis of paraganglioma (20x).

A true cut biopsy was performed and sent for frozen as well as permanent section analysis. Histologic examination revealed a well-circumscribed neoplasm composed of epithelioid chief cells with eosinophilic cytoplasm arranged in a nested pattern and peripherally surrounded by more spindle-shaped sustentacular cells. These cell nests, referred to as “zellballen”, are characteristic of paragangliomas (Figure 4). The neoplasm was histologically benign, with minimal pleomorphism, no evidence of invasion, and low mitotic activity. Frozen section diagnosis was rendered as “consistent with paraganglioma”. Immunohistochemical stains on the permanent section specimen, including chromogranin, synaptophysin, and S100, confirmed the neuroendocrine origin of this neoplasm (Figure 5). As a result, a final pathologic diagnosis of “extraadrenal paraganglioma” was issued.

**Figure 6:**
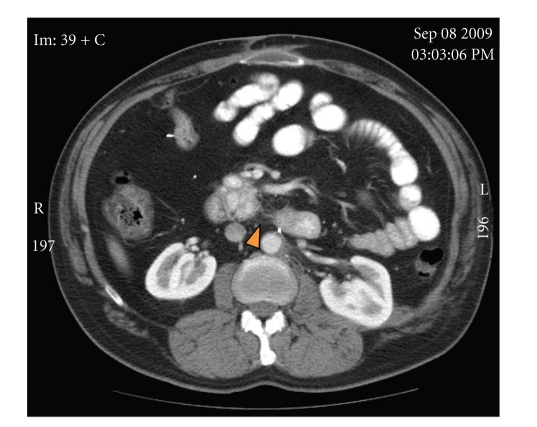
Axial contrast enhanced CT abdomen. Two years imaging follow-up shows interval decrease in size of the paraganglioma. Perilesional fat stranding is related to prior surgical procedure and radiation therapy (arrowhead).

The patient underwent radiation treatment receiving a total dose of 3960 gray divided into 22 fractions from October 17, 2006 to November 16, 2006. Repeat CT imaging 2/2007 demonstrated no significant interval change of the paraganglioma. Follow-up CT 7/2007 showed continued stability. Repeat PET 7/2007 demonstrated the retroperitoneal paraganglioma minimally smaller compared to 7/2006. On surveillance CT imaging 5/2008 the patient was noted to have a solid left renal lesion without evidence of distant metastasis. After consultation with Urology the patient underwent a partial left nephrectomy in 7/2008. Frozen diagnosis was positive for renal cell carcinoma with a negative surgical margin. Surveillance CT imaging on 9/2009 demonstrated a mildly smaller retroperitoneal paraganglioma without evidence of recurrence or spread of either primary (Figure 6).

## 3. Discussion

Primary ret roperitoneal neoplasms are rare benign and malignant mesenchymal tumors that arise in the retroperitoneum, outside of the major organs [1]. Paragangliomas are extra-adrenal pheochromocytomas that arise from chromaffin cells in the sympathetic (localized in retroperitoneum and thorax) or parasympathetic (next to aortic arch, neck, and skull base) neural paraganglia [2]. They account for 10% of adult pheochromocytomas. About 70% of sympathetic paragagliomas are intraabdominal, usually found in the perinephric and paraaortic spaces. The remaining 30% are located in the chest. Malignant retroperitoneal paragangliomas range from 30% to 50% [3]. Paragangliomas metastasize approximately in 20% to 42% of the cases. Dissemination can be hematogenous or through the lymphatic system, with the most common site of metastasis being the regional lymph nodes, bone, lung, and liver. Because benign and malignant paragangliomas have the same histological appearance, the best predictor for outcome is metastasis or recurrence [2].

Functional paragangliomas secrete norepinephrine and normetanephrine and account for 30–60% of the tumors [3]. If a secretory tumor is present, the patients undergo paroxysmal episodic hypertension, as well as the typical triad of symptoms associated with pheochromocytomas: palpitations, headache, and profuse sweating. The nonsecretory type most commonly presents as abdominal pain or mass [4]; a large proportion of these tumors are incidentally discovered in normotensive patients during imaging evaluation for other reasons [5].

The diagnosis is usually established with high urine catecholamine metabolites, VMA, and metanephrine levels [4]. Once the diagnosis of chromaffin tumor is established, the next step is to determine the extension of the disease. The imaging modality of choice for primary tumor evaluation and staging is a CT of the thorax, abdomen, and pelvis. If no lesion is found, further imaging of the organ of Zuckerland and the bladder is performed. CT imaging demonstrates 93–100% sensitivity for localizing adrenal tumors, and 90% for extra-adrenal tumors [4]. On CT, retroperitoneal paraganglioma appears as a hypervascular mass. Areas of intralesional hemorrhage and necrosis can be frequently seen as the tumor enlarges. They are commonly located in the para-aortic region, and they may be confused with other retroperitoneal tumors, especially pancreatic tumors (Figure 3) [6]. MRI is more sensitive than CT in detecting extra-adrenal tumors. Scintigraphy with 123-I labeled MIBG offers superior specificity than CT and MRI imaging [2]. 

Genetic disorders involving mutations within the succinate dehydrogenase B and D units (SDHB, SDHD) and the von Hippel-Lindau (VHL) gene places an increased risk in the development of extra-adrenal paragangliomas and adrenal pheochromocytomas, respectively [7]. Development of additional primary neoplasms has also been described, including renal cell carcinoma and thyroid cancers [8].

The possibility for malignant transformation of paragangliomas makes surgical excision the treatment of choice. Radiation therapy has been advocated for patients who cannot undergo surgery or for unresectable tumors [7]. Aggressive surgery is mandatory to obtain disease free survival. Therapy with radionucleotides may be used for tumors exhibiting uptake on diagnostic scan [4]. Octreotide can be used for treatment of inoperable paragangliomas [9]. Tumor recurrences can also be successfully excised surgically with low morbidity [10].

In conclusion, retroperitoneal paragangliomas are rare tumors, mostly benign with good prognosis, but can be locally invasive and metastasize as well. They can present with pain, mass or hypertensive episodes. Patients should be initially evaluated with catecholamine levels, followed by CT or MRI to locate the primary lesion. Surgical excision remains the mainstay of treatment, although advanced disease and prominent vascularity can at times make excision difficult or impossible.
